# Long-term Prognosis of Pediatric Ocular Disease

**DOI:** 10.14789/jmj.JMJ23-0040-R

**Published:** 2024-03-28

**Authors:** TOSHIYUKI YOKOYAMA

**Affiliations:** 1 Department of Ophthalmology, Juntendo University Nerima Hospital, Tokyo, Japan; 1 Department of Ophthalmology, Juntendo University Nerima Hospital, Tokyo, Japan

**Keywords:** pediatric ocular disease, congenital cataract, congenital corneal opacity, Peter's anomaly, limbal dermoid

## Abstract

Several problems differentiate the treatment of children, especially those with congenital ocular disease, from adults, including the absence of complaints and the complication of systemic diseases. However, the most challenging is the continuing developing anatomical and functional development and immaturity in children. Consequently, the timing of disease onset and treatment can greatly affect the prognosis, and the prognosis cannot be confirmed without long-term follow-up periods.

The prognosis for unilateral congenital cataract is very poor. However, some cases achieved good vision with successful refractive correction and amblyopia therapy, suggesting that long-term parental enthusiasm and adherence are important for the visual prognosis.

Penetrating keratoplasty is rarely performed in children, and outcomes at our hospital have been extremely poor for congenital corneal opacity over the past 28 years. The visual prognosis is also poor for large limbal dermoids approaching the center of the cornea, which did not respond to preoperative amblyopia therapy. Consequently, early excision, lamellar keratoplasty, wearing of hard contact lenses, and amblyopia therapy were considered necessary.

Treatment of pediatric ocular disease should consider the pros and cons, methods, and timing, especially the development of the pediatric eye and the time of onset of the disease.

## Introduction

Pediatric ocular diseases, especially congenital ocular diseases, pose some distinctive problems that do not occur in the adult disease, including the absence of complaints, and complications by systemic abnormalities which hinder examination, diagnosis, and treatment. The biggest challenge is that children are still developing anatomically and functionally, and are immature. Consequently, the timing of disease onset and treatment is critical to the prognosis. Furthermore, the prognosis, including the development of complications, cannot be determined without long-term follow up.

## Development of the pediatric eye

The anatomical changes in the eye include the lengthening of the ocular axis from 16 mm at birth to 22 mm at one year and 24 mm in the adult, and nearly tripling of the volume of the eye associated with this increase. Therefore, the length of the ocular axis increases rapidly during the first year of life, after which the anterior part of the eye does not change much, but the posterior part of the eye continues to grow even after the age of 10 years. As the ocular axis elongates, the cornea is rapidly flattened at 6 months^1)^.

Visual acuity in children is about 0.02 at birth and reaches 1.0 after the age of 3 years^2)^. Visual acuity only develops during a limited period in childhood. Visual sensitivity is not very high in the first 2 months of life, is high from 3 months to 2 years, and then decreases until about 8 years^3)^. However, the prognosis for vision with unilateral congenital cataract is poor even if surgery is performed within a few months of birth, whereas amblyopia therapy is effective even in older children.

## Congenital cataract

The prognosis for unilateral congenital cataract is very poor. Surgery is needed within a few months to achieve good vision, but specialist consultation is not always possible at that time^4)^. Cataract surgery is a procedure to remove the cloudy lens, so the surgery results in strong hyperopia. Intraocular lenses (IOLs) are inserted in adults, but the power of the IOL is calculated based on the axial length and the corneal curvature, which change significantly during childhood growth, and so correct selection of the lens power is difficult.

Refractive correction can be achieved with eyeglasses or contact lenses in the absence of IOLs. However, such eyeglasses require lenses with very high power resulting in aniseikonia, in which the object seen appears larger than the object seen in the other eye. For this reason, correction with contact lens is necessary. However, these lenses also have special power and are difficult to care for. Furthermore, strict occlusion therapy is needed after correcting hyperopia as treatment for amblyopia. However, occlusion treatment is also quite difficult and the duration of occlusion is less than half of the recommended time^5)^. Considering these factors, IOLs are thought to be more advantageous than contact lenses because the hyperopia is always corrected to some extent although selection of the power remains a problem.

A large prospective study by a North American group, the Infant Aphakia Treatment Study group, has largely concluded the prognostic impacts, and advantages and disadvantages of IOL implantation. The prognosis of 114 patients with unilateral congenital cataracts treated surgically before 6 months of age, half with contact lens and half with IOL implants, was prospectively studied. At one year of age, no difference in visual acuity was found between the two groups. However, there were 2.5 times more intraoperative complications, 6 times more additional surgeries, and 3 times more adverse events with IOLs^6)^.

Contact lenses seem to be the better choice by far, but the absence of any difference in visual acuity despite these various problems suggests that a longer study may detect differences. Comparison of the groups at age 5 years again found no difference in visual acuity. Half of the participants had visual acuities of less than 0.1, and most had strabismus and high frequency of glaucoma^7)^. Visual acuity did not differ between the contact lens and IOL groups as expected at age 10 years, when visual development was almost complete. Forty-four percent of patients had visual acuity of 0.1 or less. The final conclusion was that IOL implantation was neither beneficial nor detrimental to visual acuity^8)^.

**Figure 1:**
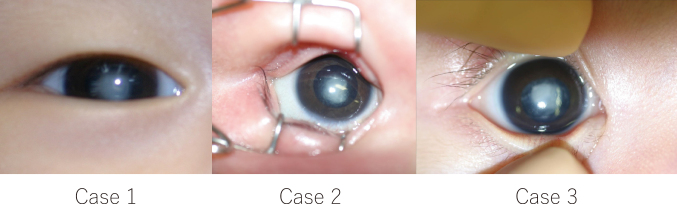
Preoperative photographs of the anterior segment of the eyes showing cataracts with dense central opacities. *Left*: Case 1, *center*: Case 2, *right*: Case 3.

Three patients with unilateral congenital cataracts were treated surgically early in life, before 6 months, and followed up for more than 7 years, consistent with the previous study, in Juntendo University Nerima Hospital. The preoperative severity of the cataracts was similar in all three patients, as shown in Figure 1. However, the final visual acuity was 0.07 in Case 1, 0.7 in Case 2, and 1.2 in Case 3 (Table 1).

**Table 1:** Summary of three cases of unilateral congenital cataract

Case No.	Age at first visit (days)	Age at surgery (days)	Compliance of occlusion	Compliance of wearing glasses or contact lenses	Eye position	Final VA (age)	Best VA (age)	Postoperative complication
1	108	116	Not good	Not good	Esotropia	0.07 (10 years 5 months)	0.4 (3 years 1 month)	None
2	40	40	Good	Not good	Exotropia	0.7 (7 years 7 months)	0.7 (5 years 7 months)	Temporal choroidal detachment
3	39	39	Good	Good	Esotropia	1.2 (9 years)	1.2 (6 years 9 months)	Synechia iridis posterior

VA: visual acuity.

Case 1 was first diagnosed after 3 months. The mother had earlier noticed leukocoria in her right eye and complained at the one-month checkup, but no close examination was done. The surgery was performed within 10 days of the first visit as an associate emergency. She had a maximum visual acuity of 0.4 at age 3 years. However, the patient could not tolerate wear contact lenses or occlusion well, and she developed strabismus within 2 years, so we abandoned binocular vision and instructed her to wear glasses, but she was not able to do that very well either.

Case 2 could not or did not wear contact lenses, but exotropia appeared at 2 years after surgery, so the patient wore both glasses and contact lenses, and did her best to occlude her healthy eye, which enabled her to achieve visual acuity up to 0.7.

Case 3 was able to continuously wear contact lenses and strictly occlude her healthy eye. It is very rare for a child to achieve 1.2 vision. The reason is that the mother was a nurse who still works at our hospital and had previously worked in the ophthalmology department. Consequently, her mother understood the importance of refractive correction and occlusion therapy to achieve good vision.

## Congenital corneal opacity

Congenital corneal opacity is a rare disease, found in only two to three of every 100,000 live births^9, 10)^, of which 40% are Peter's anomaly, followed by dermoid and sclerocornea, and includes corneal dystrophy, which is rarely seen, metabolic disorders such as Hurler's syndrome, which is also rarely seen, congenital glaucoma, and forceps delivery trauma.

The main treatment for corneal opacity is keratoplasty. A review of all keratoplasties performed at our hospital over a 28-year period from 1981 to 2008 revealed that only 25 children underwent penetrating keratoplasty, equivalent to about 1% of the adult population. Transparent grafting is reasonably successful for keratoconus and herpetic keratitis, but very poor for congenital conditions such as Peter's anomaly and congenital corneal staphyloma, as shown in Table 2. Furthermore, visual acuity is very poor in patients with Peter's anomaly even if keratoplasty was successful, compared to acquired conditions such as keratoconus or herpes keratitis (Table 3). Poor postoperative results are widely reported with long-term Peter's anomaly^11-13)^. However, good outcomes are known^14, 15)^, various poor prognostic factors such as vascular invasion, glaucoma, and lens abnormalities have been identified, and new methods of treatment using Descemet's stripping automated endothelial keratoplasty have been reported^16)^. In any case, early surgery and thorough low vision treatment are necessary.

**Table 2:** Penetrating keratoplasty under age 15 years and rates of clear grafts

	Number of eyes	Clear grafts	Rate of clear graft (%)
Peter’s anomaly	9	2	22.2
Keratoconus	9	8	88.9
Herpetic keratitis	6	4	66.7
Corneal staphyloma	1	0	0
Total	25	14	56.0

**Table 3:** Visual acuity with clear graft

	Preoperative	Postoperative
Peter’s anomaly	1: ?	0.01
	2: ?	0.01
Keratoconus	0.01-0.1	≥0.8 except one with 0.1 vision
Herpetic keratitis	1: 0.02	0.7
	2: 0.01	0.1
	3: 0.02	0.8
	4: 0.01	0.4

**Figure 2:**
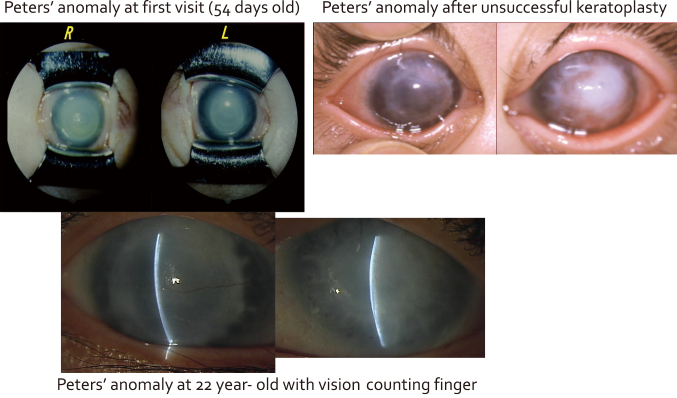
Photographs of the anterior segment of the eyes at the time of initial examination at age 54 days (*upper left*), after unsuccessful keratoplasty (*upper right*), and at age 22 years (*lower*) in a patient with Peter’s anomaly with clouded grafts.

I present a case of bilateral Peter's anomaly with early onset glaucoma. The patient was one month old when she was first seen at our hospital, and had already been treated for glaucoma, which is a complication of Peter's anomaly, at another hospital. She had high intraocular pressure after coming to our hospital, so she underwent a trabeculotomy to control the intraocular pressure and penetrating keratoplasty in her left eye at age 6 months and in her right eye at age 1 year 3 months. However, the graft in the left eye became cloudy at 8 months and the right graft became cloudy at 1 month after keratoplasty. Subsequently, she underwent 3 transplants, cataract extraction, 2 trabeculotomies, and 3 trabeculectomies in her left eye, and 2 trabeculotomies and 2 trabeculectomies in her right eye. Figure 2 shows the anterior segment of the eyes at the time of initial examination, after unsuccessful keratoplasty, and at age 22 years. Her visual acuity was finger counting in both eyes at her last visit, which was considered a better outcome than no surgery although the visual prognosis was poor.

We have experienced 60 cases of pediatric lamellar keratoplasty in 18 years. Most cases were limbal dermoid and the prognosis was reasonable (Table 4). Limbal dermoids are congenital choristomatous lesions consisting of ectodermal and mesodermal elements and appear as yellowish-white, dome-shaped masses, usually at the inferotemporal limbus of the eye^17)^. Dermoids can affect the visual acuity by inducing regular or irregular astigmatism and hyperopic anisometropia^18)^. However, if the dermoid or associated lipid infiltration encroach on the visual axis, the visual prognosis is very poor^18, 19)^. Therefore, we investigated whether the degree of encroachment or encroachment index (EI) of the dermoid tumor can be used as a prognostic factor of visual acuity after lamellar keratoplasty.

**Table 4:** Lamellar keratoplasty under age 15 years

	Number of eyes	Number of clouded grafts
Limbal dermoid	45	2
Herpetic keratitis	2	0
Corneal ulcer	2	0
Gelatinous drop-like corneal dystrophy	2	2
Others	9	5
Total	60	9

**Figure 3:**
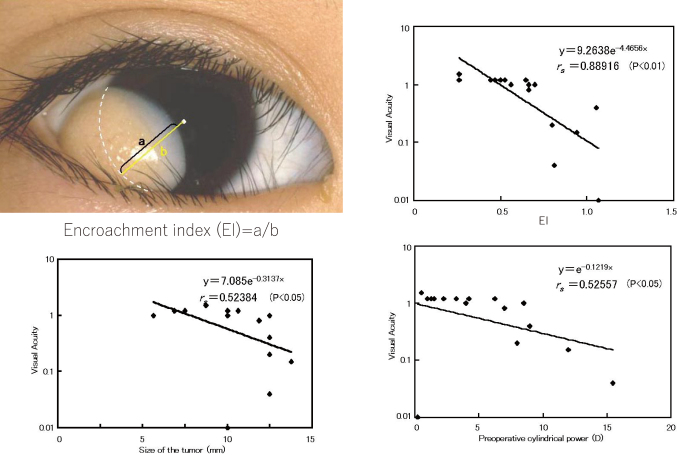
*Upper left*: Photograph of a relatively large limbal dermoid tumor to demonstrate calculation of the encroachment index (EI). The EI is the ratio of the distance from the estimated limbus to the papillary edge of the tumor (a) divided by the distance from the estimated limbus to the center of the pupil (b) along the axis of the tumor. *Upper right*: Correlation between visual acuity and EI. *Lower left*: Correlation between visual acuity and tumor size. *Lower right*: Correlation between visual acuity and preoperative cylindrical power.

The medical records of eight boys and nine girls with limbal dermoids were reviewed (Table 5). Age at surgery, amblyopia therapy, preoperative cylindrical power, tumor size (largest diameter), and visual acuity at the final visit were recorded. The EI of the limbal dermoids was calculated as the ratio of the distance from the estimated (because the limbus is covered by the tumor) limbus to the papillary edge of the tumor divided by the distance from the estimated limbus to the center of the pupil (Figure 3 *upper left*). A value of 1.0 indicated that the tumor was at the center of the pupil, and <1.0 that the tumor had not reached the center. Values >1.0 indicated that the tumor had spread past the center of the pupil. The dermoid tumor was unilateral in all cases, and the follow-up time ranged from 6 months to 244 months. Patients with multiple tumors, other ocular disease or mental retardation were excluded.

**Table 5:** Summary of 17 cases

Case No.	Amblyopic therapy	Preoperative cylindrical power (D)	Age at surgery (years)	Size of the tumor (mm)	ECRs	Visual acuity
1	－	0.5	6	7	0.26	1.5
2	＋	2.25	8	5.5	0.26	1.2
3	－	1	5	8	0.44	1.2
4	＋	1.5	5	6	0.47	1.2
5	－	1.25	12	8.5	0.50	1.2
6	＋	4.25	10	8	0.50	1.2
7	＋	3.25	9	8.5	0.53	1.2
8	＋	4	9	4.5	0.56	1
9	＋	6.25	13	8	0.65	1.2
10	＋	4	10	8	0.67	1
11	＋	7	6	9.5	0.67	0.8
12	＋	8.5	7	10	0.70	1
13	＋	8	6	10	0.80	0.2
14	＋	15.5	4	10	0.81	0.04
15	＋	12	3	11	0.94	0.15
16	＋	9	1	10	1.06	0.4
17	＋	0.25	3	8	1.07	0.01

D: diopters.

Fifteen patients received single lamellar keratoplasty with good cosmetic results. One patient (17) had 3 operations because of graft melting and another patient (15) had two operations because the graft was too small. Six of the 17 patients had auricular appendages and were diagnosed with Goldenhar's syndrome.

Correlation coefficient of the final visual acuity was 0.889 with the EI (*P* = 1.87 x 10^-^^6^; Figure 3 *upper right*), 0.524 with the tumor size (*P* = 0.031; Figure 3 *lower left*), and 0.526 with the preoperative cylindrical power (*P* = 0.03; Figure 3 *lower right*; Spearman's rank correlation coefficient). Five patients had final visual acuity of less than 0.7, and four had had surgery after age 3 years. Tumor larger than 10 mm, cylindrical power >7.0 diopters, and EI >0.8 were risk factors for poor visual acuity. However, Case 12 with a large 10 mm tumor, 8.5 diopters cylindrical power, and EI of 0.7 had good final visual acuity, and Case 17 with a relatively small tumor, low cylindrical power, and EI of 1.07 had poor visual acuity. Most importantly, all patients with EI >0.8 had poor visual acuities. The EI is easy to calculate and the high correlation with the final visual acuity after lamellar keratoplasty indicates that corneal extension is the most important factor for the visual prognosis and EI can used as a prognostic factor for patients indicated for lamellar keratoplasty for limbal dermoid.

**Figure 4:**
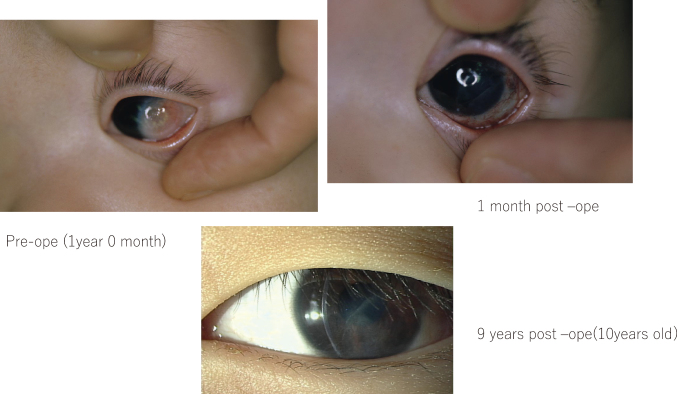
A representative patient with large limbal dermoid treated by early surgery and correction with hard contact lenses and occlusion therapy. *Upper left*: Preoperative photograph (age 1 year 0 month). *Upper right*: Postoperative photograph after 1 month. *Lower*: postoperative photograph after 9 years (age 10 years).

These large dermoids would not respond to amblyopia therapy, so we decided to treat as soon as possible and then correct amblyopia with hard contact lenses and occlusion therapy. The representative case shown in Figure 4 had a limbal dermoid extending to the center of the cornea. In general, surgery is performed after amblyopia treatment, mostly at age 5 years or later, but this patient was treated at age 1 year, and after wearing hard contact lenses with occlusion therapy, she was able to achieve 0.4 vision.

In conclusion, treatment should consider that the child patient is still developing, and the prognosis will not be confirmed until after a longer follow-up period.

## Funding

No funding was received.

## Author contributions

The author read and approved the final manuscript.

## Conflicts of interest statement

The author declares that there are no conflicts of interest.
